# *Origanum vulgare* leaf extract protects mice bone marrow cells against ionizing radiation

**Published:** 2016

**Authors:** Reza Ghasemnezhad Targhi, Vahid Changizi, Farhang Haddad, Mansour Homayoun, Shokouhozaman Soleymanifard

**Affiliations:** 1 *Department of Radiology, School of Allied, Tehran University of Medical Sciences, Tehran, Iran*; 2 *Department of Biology, Faculty of Sciences, Ferdowsi University of Mashhad, Mashhad, Iran*; 3 *Department of Anatomy, Faculty of Medicine, Isfahan University of Medical Sciences, Isfahan, Iran*; 4 *Department of Medical Physic, Faculty of Medicine, Mashhad University of Medical Sciences, Mashhad, Iran*; 5 *Medical Physics Research Center, Mashhad University of Medical Sciences, Mashhad, Iran*; 6 *Medical Physics Department, Omid hospital, Mashhad, Iran*

**Keywords:** *Radioprotective agents*, *Micronucleus*, *Bone marrow cells*, *Whole-body irradiation*, *Origanum vulgare*

## Abstract

**Objective:**

Ionizing radiation produces free radicals which induce DNA damage and cell death. *Origanum vulgare *leaf extract (OVLE) is a natural compound and its capability of scavenging free radicals and its antioxidant activity have been demonstrated by many researchers*.* In this study, using micronucleus assay, radioprotective effect of OVLE against clastogenic and cytotoxic effect of gamma irradiation has been investigated in mice bone marrow cells.

**Materials and Methods:**

OVLE was injected intraperitoneally to the BALB/c mice 1hr prior to gamma irradiation (3Gy) at the doses of 100 and 200 mg/kg. Twenty four hours after irradiation or treatment, animals were killed and smears were prepared from the bone marrow cells. The slides were stained with May Grunwald–Giemsa method and analyzed microscopically. The frequency of micronucleated polychromatic erythrocytes (MnPCEs), micronucleated normochromatic erythrocyte (MnNCEs) and cell proliferation ratio PCE/PCE+NCE (polychromatic erythrocyte/polychromatic erythrocyte + normochromatic erythrocyte) were calculated.

**Results:**

The results showed that gamma irradiation (3Gy) increased the frequency of MnPCEs, MnNCEs and reduced the PCE/PCE+NCE ratio in mice bone marrow compared to the non-irradiated control group (p<0.0001). Injection of OVLE significantly reduced the frequency of MnPCEs (p<0.0001) and MnNCEs (p<0.05) and increased the PCE/PCE+NCE ratio as compared to the irradiated control group (p<0.05).

**Conclusion:**

It seems that OVLE with its antioxidant properties and its capability of scavenging free radicals and reactive oxygen species can reduce the cytotoxic effects of gamma irradiation in mice bone marrow cells.

## Introduction

Ionizing radiation can produce reactive free radicals (e.g. hydroxyl radicals, hydrogen radicals) and a toxic substance (i.e. hydrogen peroxide) by passing through living tissues and interacting with water in the cells. Free radicals interact with critical macromolecules, such as DNA and proteins, and induce cell damage or cell death (Karbownik and Reiter 2000 ▶). On the other hand, some cellular components (thiols) which have the ability to scavenge the free radicals confer protection against harmful effects of radiation (Hosseinimehr, 2007 ▶). Artificial radioprotective agents are chemical compounds or natural products that are administrated before irradiation to reduce radiation injuries (Hosseinimehr, 2007 ▶). The synthetic thiol compounds which are highly effective in reducing radiation-induced lethality, have been widely studied (Brown et al., 1982 ▶, Held and Biaglow 1994 ▶, Cassatt et al., 2002 ▶). However, at efficient concentrations, they are toxic and cause some side effects. For example amifostine, the only thiol compound approved by the Food and Drug Administration (FDA) (Brown et al., 1982 ▶, Cassatt et al., 2002 ▶) causes nausea, vomiting and hypotension. In recent years, radioprotective agents with a new action have been investigated; particularly, compounds that can affect hematopoietic stem cell regeneration have attracted significant interest (Whitnall et al., 2000 ▶, Landauer et al., 2003 ▶). Herbal compounds act as antioxidants/immune stimulants, and are another strategy for the development of radioprotective agents with low toxicity (Hosseinimehr, 2007 ▶). Among different plants, *Origanum vulgare* is rich in powerful antioxidants, and is able to neutralize free radical activity, and reduce the secretion of NO (nitric oxide) (Faleiro et al., 2005 ▶). *O. vulgare* is native to Europe and came to the United States at the beginning of the 20th century. Many *in vitro* and *in vivo* studies showed that *O. vulgare* extract has antibacterial (Lambert et al., 2001 ▶), antifungal (Sivropoulou et al., 1996 ▶), antioxidant (Kulisic et al., 2004 ▶; Karakaya et al., 2011 ▶; Ceker et al., 2012 ▶), anti-carcinogenic (Teissedre and Waterhouse, 2000 ▶) and anti-mutagenic activities (Ipek et al., 2005 ▶; Mezzoug et al., 2007 ▶; Özbek et al., 2008 ▶). It is suggested that rosmarinic acid, flavonoids, carvacrol and thymol that are present in the *O. vulgare* extracts are responsible for the above-mentioned properties (Burt et al., 2007 ▶; Lee et al., 2008 ▶; De Martino et al., 2009 ▶). Also, it is likely that the strong antioxidant property of this plant is related to its phenolic compounds (e.g. carnozol, rozmanol, rosamaridiphenol, and rosmarinic acid) (Lagouri and Boskou, 1996 ▶). Rosmarinic acid and hydroxycinnamic acid have been demonstrated to possess strong antioxidant activity (Larson, 1988 ▶; Chen and Ho, 1997 ▶). Some research proved that antioxidant activity of *O. vulgare* extracts was higher than R-tocopherol and was comparable to that of butylated hydroxyanisole (BHA) against linoleic acid oxidation (Nakatani, 1992 ▶). Radioprotective properties of plant extracts are mainly studied by assessing their ability to reduce radiation-induced chromosomal aberrations and micronuclei formation (Hosseinimehr, 2007 ▶).

In the present study, the radioprotective effect of OVLE against gamma radiation was investigated by the micronucleus assay in BALB/c mice.

## Materials and Methods

**Plant extract**

*O. vulgare* leaves were collected from the mountains of Kalat in early summer of 2014, (Khorasan Razavi, Iran) and were identified by the botanists. Then, 100 g of fresh *O. vulgare *leaves were dried in shadow at room temperature (No color change was observed), powdered and soaked in ethyl alcohol 70% at room temperature for 48 hr. During this time, the mixture was stirred intermittently. Finally, the prepared solution was filtered using filter paper and kept in Bain Marie at 40°C for 72 hr to obtain dried powder.

**Animals**

Adult male BALB/c mice (6-8 weeks old; weighing 25-30 g) were purchased from Pasteur Institute (Tehran, Iran). The animals were maintained in the animal house and had free access to standard mouse pellet and water *ad libitum*. All animals were kept at 22± 2°C under controlled light condition (light: dark, 12 hr:12 hr) .

**Treatment**

One hour before irradiation, *OVLE* was dissolved in distillated water and at the doses of 100 and 200 mg/kg were injected intraperitoneally to the experimental animals. The control group received the same volume of distillated water.

**Irradiation**

The mice were irradiated by a cobalt-60 gamma radiation source (Teratron 780, Canada, at the dose rate of 54 cGy/min) at room temperature (23± 2°C). The mice were exposed to 3 Gy whole body irradiation, while they were placed in ventilated Plexiglas cages and the source-to-skin distance was 70 cm. 

**Experimental Design**

Forty two mice were randomly divided into six groups (seven mice in each group):

Group I (Control): Animals received distillated water intraperitoneally.

Group II: Animals received 100 mg/kg OVLE intraperitoneally.

Group III: Animals received 200 mg/kg OVLE intraperitoneally.

Group IV: Animals were exposed to 3Gy gamma radiation.

Group V: Animals received 100 mg/kg OVLE intraperitoneally and after 1 hr were exposed to 3 Gy whole body gamma irradiation.

Group VI: Animals received 200 mg/kg OVLE intraperitoneally and after 1 hr were exposed to 3 Gy whole body gamma irradiation.

Twenty four hour after irradiation or treatment, the animals were sacrificed and both femurs were removed for micronucleus assay.

**Micronucleus assay**

The mice bone marrow micronucleus test was carried out according to the method described by Schmid (Schmid, 1975 ▶). Twenty four hours after irradiation or treatment, the animals were sacrificed and both femurs were removed. The bone marrow from femurs was flushed in the form of a fine suspension into a centrifuge tube containing fetal bovine serum (FBS). The cells were collected by centrifugation at 1000 rpm for 10 min at 4 °C. Bone marrow smears were prepared and the slides were kept at room temperature. After 24 hr air-drying, smears were fixed with methanol and stained with May-Grunwald/ Giemsa (Merck, Darmstadt, Germany). According to this method, polychromatic erythrocytes (PCEs) and normochromatic erythrocytes (NCEs) were stained reddish-blue and orange, respectively, while nuclear material was dark purple. For each experimental group, seven mice were used and a total of seven microscopic slides were prepared. Then, 1000 PCEs were scored per slide to determine the percentage of micronucleated polychromatic erythrocytes (MnPCEs), micronucleated normochromatic erythrocytes (MnNCEs), and ratio of PCE/PCE+NCE. 

**Statistical analysis**

Statistical analysis was performed using SPSS 16. All data were distributed normally; therefore, One-way ANOVA analysis and Turkey's HSD test were used for multiple comparisons of data. A p< 0.05 was considered statistically significant. 

## Results

**Effect of gamma irradiation on mice bone marrow cell**

**Figure 1:**
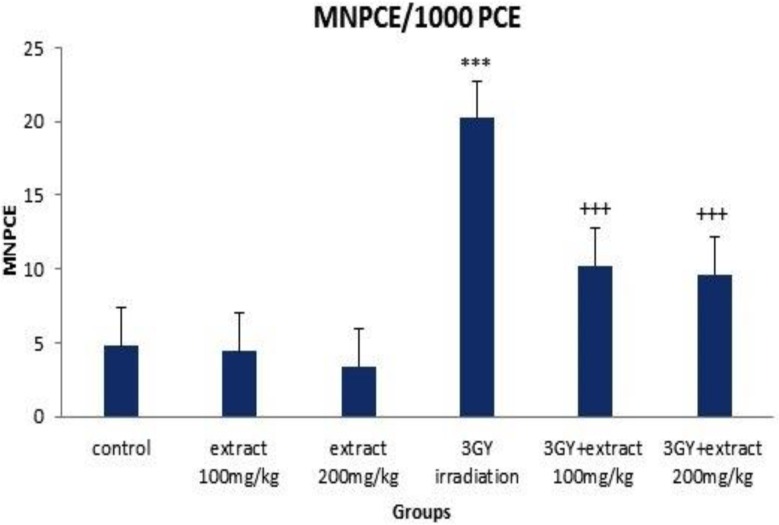
Mean number of micronucleated cells per 1000 polychromatic erythrocytes cells (MNPCE/1000 PCE) in different groups. Error bars indicate standard errors of mean values. *** p< 0.001 as compared to control group, +++ p< 0.001 as compared to the 3GY gamma-irradiated group. Control, extract 100 mg/kg, and extract 200 mg/kg indicate the sham irradiated groups which received distilled water and 100 and 200 mg/kg OLVE, respectively.

**Figure 2:**
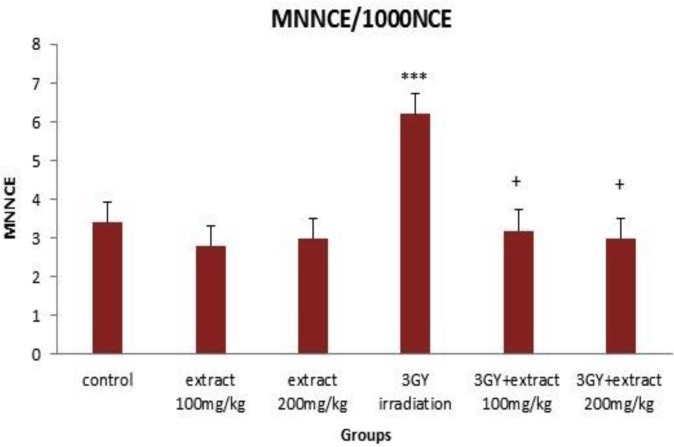
Mean number of micronucleated cells per 1000 normochromatic erythrocyte cells (MNNCE/1000 NCE) in different groups. Error bars indicate standard errors of mean values. *** p <0.001 as compared to control group, + p< 0.05 as compared to the 3GY gamma-irradiated group.

**Figure 3:**
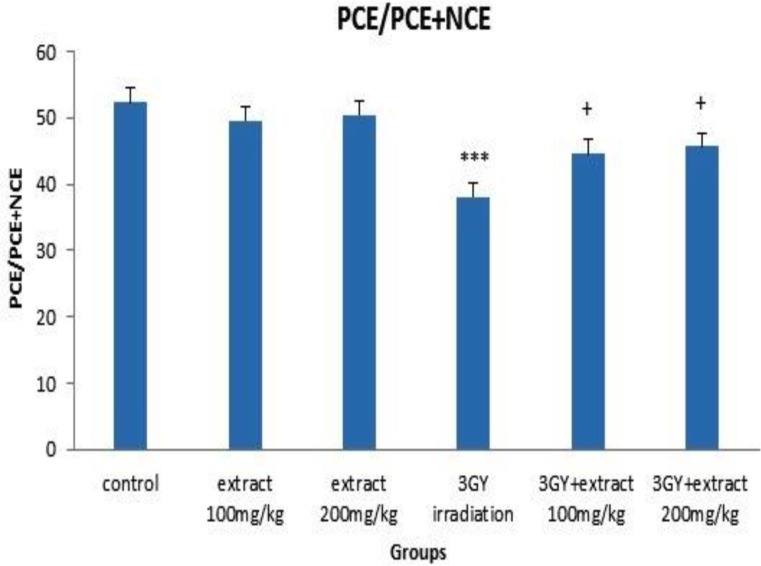
The cell proliferation ratio (PCE/PCE+NCE) in mice bone marrow cells. Error bars indicate standard errors of mean values. *** p< 0.001 as compared to control group, + p< 0.05 as compared to the 3Gy of ^60 ^Cogamma rays.

The frequency of MnPCE and MnNCE significantly increased in the group of 3Gy gamma irradiation as compared to the control group (p< 0.00001). The increased frequency of MnPCE was remarkably higher than MnNCE. The cell proliferation ratio (PCE/PCE+NCE) significantly decreased by 3Gy gamma irradiation (p<0.00001) (Figure 1-3). The data revealed that 3Gy gamma irradiation induced genotoxicity and cytotoxicity in mice bone marrow cells.

**Effect of OVLE on mice bone marrow cell**

OVLE at the doses of 100 and 200 mg/kg was injected to animals and after 24 hr, the frequency of MnPCE, MnNCE and PCE/PCE+NCE were evaluated in bone marrow cells. Injection of OVLE alone, without irradiation, did not change the frequency of MnPCE and MnNCE and the ratio of PCE/PCE+NCE was not significantly different from the control group (p>0.05). These results indicated that OVLE did not have any clastogenic and cytotoxic effects on mice bone marrow cells (Figure 1-3).

**Effect of OVLE against gamma irradiation**

Compared to the group which received 3 Gy irradiation (without OVLE), and as a result of 100 mg/Kg OVLE administration prior to irradiation, 49.50% (p<0.0001) and 48.38% (p<0.05) reductions were observed for MnPCE and MnNCE, respectively. Corresponding reduction as a result of administration 200 mg/Kg OLVE before irradiation were 52.47% (p<0.0001) and 51.61% (p<0.05). PCE/PCE+NCE (p<0.05) compared to the 3Gy gamma-irradiated group (without OVLE) increased up to 15.22% (p< 0.05) and 17.88% (p< 0.05) as a result of administration of 100 and 200 mg/kg, respectively (Figure 1-3). 

3Gy irradiation group was treated with only 3 Gy^60 ^Cogamma rays.

3Gy+extract 100 mg/kg and 3Gy+extract 200 mg/kg indicate the groups which were respectively treated with 100 and 200 mg/kg OVLE 1hr prior to irradiation.

Control, extract 100 mg/kg, extract 200 mg/kg indicate the sham irradiated groups which received distilled water and 100 and 200 mg/kg OLVE, respectively. 3Gy irradiation group was treated with only 3 Gy 60 Co gamma rays.

3Gy+extract 100 mg/kg and 3Gy+extract 200 mg/kg indicate the groups which were respectively treated with 100 and 200 mg/kg OVLE 1hr prior to irradiation.

Control, extract 100 mg/kg, extract 200 mg/kg indicate the sham irradiated groups which received distilled water and 100 and 200 mg/kg OLVE, respectively.

3Gy irradiation group was treated with only 3 Gy^60 ^Cogamma rays.

3Gy+extract 100mg/kg and 3Gy+extract 200mg/kg indicate the groups which were respectively treated with 100 and 200 mg/kg OVLE 1hr prior to irradiation.

## Discussion

In the present study, the radioprotective effect of OVLE was investigated. We observed that OVLE significantly reduced the number of MnPCE and MnNCE induced by gamma radiation in mice bone marrow. It also increased the ratio of PCE/PCE+NCE, which was reduced by radiation. In line with our results, protective effect of OVLE has been reported in different studies. Ceker et al. (2012) ▶ showed that OVLE was able to decrease the frequencies of micronucleus and sister chromatic exchange induced by Aflatoxin B1. Arami showed that treatment of human lymphocytes with 25, 50 and 100 μg/ml of *O. vugare* reduced the frequency of micronuclei (MN) induced by Radioiodine (^131^I) (Arami et al., 2013 ▶). Kapiszewska demonstrated that pre-treatment of lymphocytes with OVLE and several other plants inhibited oxidative DNA damage induced by hydrogen peroxide (Kapiszewska et al., 2005 ▶).

It has been shown that antioxidant agents can neutralize free radical species induced by radiation and consequently inhibit their side effects (Hosseinimehr, 2007 ▶). The radioprotective effects of *O. vugare* are attributed to its antioxidant activities. This herbal agent contains phenolic compounds, such as thymol and carvacrol, which are rich in OH groups and able to scavenge free radicals (Roofchaee et al., 2013 ▶). Several studies have shown that thymol and carvacrol have chemoprotective and radioprotective effects against toxicity and genotoxicity induced by chemical agents and ionizing radiation (Archana et al., 2009 ▶;Vicuña et al., 2010 ▶). Using DPPH (1,1-diphenyl-2-picrylhydrazyl-free radicals) method, it was shown that antioxidant activities of *O. vulgare* is higher than butylated hydroxytoluene (BHT), a standard antioxidant (Arami et al., 2013 ▶). Kapiszewska concluded the protective effect of *Origunam heracleoticum* is dependent on polyphenol concentrations, which efficiently scavenges the reactive radical species (Kapiszewska et al., 2005 ▶). Another mechanism was proposed by Suryakant to explain the *Origanum majorana *radioprotective activity. They showed that *Origanum* extract caused an increase in the levels of O6-methylguanine-DNA methyltransferase (MGMT) that is a DNA protective protein. They also demonstrated that *Origanum* has demethylation activity, which increases in a time-dependent manner up to a maximum of 3-fold after 72 hr of treatment (Niture et al., 2006 ▶).

Since radioprotective properties of chemicals are accompanied by toxicity, plant extracts have been considered as substitutes for chemical radioprotectors. Many investigations have been performed to explore non-toxic alternative radioprotectors. In some studies, Triphala, *Hippophae rhamnoides*, *Mangifera indica*, *Panax ginseng*, *Mentha piperita*, *Tinospora cordifolia*, *Aegle marmelos*, Naringin and Spirulina, have been injected to mice. In all cases, mortality and symptoms of radiation sickness significantly decreased in injected mice compared to control groups (Jagetia et al., 2002 ▶; Jagetia et al., 2003 ▶; Jagetia et al., 2004 ▶; Samarth et al., 2004 ▶; Lee et al., 2005 ▶; Sharma et al., 2011 ▶; Khan et al., 2014 ▶). Employing micronucleus assay, the radioprotective activity of some plants in mice bone marrow cells has been investigated (Hosseinimehr et al., 2003 ▶, Hosseinimehr et al., 2007 ▶, Hosseinimehr and Nemati, 2014 ▶)

 Our results showed that injection of 100 and 200 mg/kg OVLE, 1 hr prior to 3 Gy gamma irradiation, reduced the frequency of MNPCE and MNNCE cells and increased the cell proliferation ratio (PCE/PCE+NCE). Although there was not any statistically difference between the two doses of OVLE, the dose of 200 mg/kg was more effective (with 51.61% reduction in MnPCE). However, since OVLE has been used extensively as a herbal and additive agent, and with regards

to potential radioprotective effect, it is possible to apply higher amounts of extract in future studies to investigate the possibility of complete removal of radiation effects. 

The results of the current study, using micronucleus assay, confirmed the radioprotective activity of OVLE. Hence, OVLE is recommended to be included in human diets to prevent side effects associated with environmental and human-made radiation. However, further comprehensive *in vivo* research regarding the appropriate dose and treatment period is required to support the obtained findings. Besides, it would be beneficial to investigate other biological end points (radiation genotoxicity) to confirm the results, and remove any doubt about OVLE toxicity.

## References

[B1] Arami S, Ahmadi A, Haeri SA (2013). The radioprotective effects of Origanum vulgare extract against genotoxicity induced by 131I in human blood lymphocyte. Cancer Biother. Radiopharm.

[B2] Archana P, Rao BN, Ballal M, Rao BS (2009). Thymol, a naturally occurring monocyclic dietary phenolic compound protects Chinese hamster lung fibroblasts from radiation-induced cytotoxicity. Mutat Res Genet Toxicol Environ Mutagen.

[B3] Brown DQ, Pittock JW, Rubinstein JS (1982). Early results of the screening program for radioprotectors. Int J Radiat Oncol Biol Phys.

[B4] Burt SA, van der Zee R, Koets AP, de Graaff AM, van Knapen F, Gaastra W (2007). Carvacrol induces heat shock protein 60 and inhibits synthesis of flagellin in Escherichia coli O157: H7. J Appl Environ Microbiol.

[B5] Cassatt DR, Fazenbaker CA, Bachy CM, Hanson MS (2002). Preclinical modeling of improved amifostine (Ethyol) use in radiation therapy. Semin Radiat Oncol.

[B6] Ceker S, Agar G, Nardemir G, Anar M, Kizil HE, Alpsoy L (2012). Investigation of anti-oxidative and anti-genotoxic effects of Origanum vulgare L essential oil on human lymphocytes in vitro. J Essent Oil Bear Pl.

[B7] Chen JH, Ho C-T (1997). Antioxidant activities of caffeic acid and its related hydroxycinnamic acid compounds. J Agr Food Chem.

[B8] De Martino L, De Feo V, Formisano C, Mignola E, Senatore F (2009). Chemical composition and antimicrobial activity of the essential oils from three chemotypes of Origanum vulgare L ssp hirtum (Link) Ietswaart growing wild in Campania (Southern Italy). Molecules.

[B9] Faleiro L, Miguel G, Gomes S, Costa L, Venâncio F, Teixeira A (2005). Antibacterial and antioxidant activities of essential oils isolated from Thymbra capitata L (Cav) and Origanum vulgare L. J Agr Food Chem.

[B10] Held KD, Biaglow JE (1994). Mechanisms for the oxygen radical-mediated toxicity of various thiol-containing compounds in cultured mammalian cells. J Radiat Res.

[B11] Hosseinimehr S, Nemati A (2014). Radioprotective effects of hesperidin against gamma irradiation in mouse bone marrow cells. Brit J Radiol.

[B12] Hosseinimehr S J (2007). Trends in the development of radioprotective agents. Drug Discov Today.

[B13] Hosseinimehr SJ, Azadbakht M, Mousavi SM, Mahmoudzadeh A, Akhlaghpoor S (2007). Radioprotective effects of hawthorn fruit extract against gamma irradiation in mouse bone marrow cells. J Radiat Res.

[B14] Hosseinimehr SJ, Tavakoli H, Pourheidari G, Sobhani A, Shafiee A (2003). Radioprotective Effects of Citrus Extract Against γ;-Irradiation in Mouse Bone Marrow Cells. J Radiat Res.

[B15] Ipek E, Zeytinoglu H, Okay S, Tuylu BA, Kurkcuoglu M, Baser KHC (2005). Genotoxicity and antigenotoxicity of Origanum oil and carvacrol evaluated by Ames Salmonella/microsomal test. Food Chem.

[B16] Jagetia G, Baliga MS, Malagi K, Kamath MS (2002). The evaluation of the radioprotective effect of Triphala (an ayurvedic rejuvenating drug) in the mice exposed to γ-radiation. Phytomedicine.

[B17] Jagetia G, Venkatesh P, Baliga M (2004). "Evaluation of the radioprotective effect of bael leaf (Aegle marmelos) extract in mice. Int J Radiat Biol.

[B18] Jagetia GC, Venkatesha V, Reddy TK (Naringin). 2003. Naringin.

[B19] Kapiszewska M, Soltys E, Visioli F, Cierniak A, Zajac G (2005). The protective ability of the Mediterranean plant extracts against the oxidative DNA damage The role of the radical oxygen species and the polyphenol content. J Physio Pharmacol. Supplement.

[B20] Karakaya S, El SN, Karagözlü N, Şahin S (2011). Antioxidant and antimicrobial activities of essential oils obtained from oregano (Origanum vulgare ssp hirtum) by using different extraction methods. J Med Food.

[B21] Karbownik M, Reiter RJ (2000). Antioxidative effects of melatonin in protection against cellular damage caused by ionizing radiation. P Soc Exp Biol Med.

[B22] Khan A, Manna K, Das DK, Sinha M, Kesh SB, Das U (2014). Seabuckthron (Hippophae rhamnoides L) leaf extract ameliorates the gamma radiation mediated DNA damage and hepatic alterations. Indian J Exp Biol.

[B23] Kulisic T, Radonic A, Katalinic V, Milos M (2004). Use of different methods for testing antioxidative activity of oregano essential oil. Food Chem.

[B24] Lagouri V, Boskou D (1996). Nutrient antioxidants in oregano. Int J Food Sci Nutr.

[B25] Lambert R, Skandamis PN, Coote PJ, Nychas GJ (2001). A study of the minimum inhibitory concentration and mode of action of oregano essential oil, thymol and carvacrol. J Appl Microbiol.

[B26] Landauer MR, Srinivasan V, Seed TM (2003). Genistein treatment protects mice from ionizing radiation injury. J App Toxicol.

[B27] Larson RA (1988). The antioxidants of higher plants. Phytochemistry.

[B28] Lee S, Buber M, Yang Q, Cerne R, Cortes R, Sprous D (2008). Thymol and related alkyl phenols activate the hTRPA1 channel. Brit J Pharmacol.

[B29] Lee T-K, Johnke RM, Allison RR, O'Brien KF, Dobbs LJ (2005). Radioprotective potential of ginseng. Mutagenesis.

[B30] Mezzoug N, Elhadri A, Dallouh A, Amkiss S, Skali N, Abrini J (2007). Investigation of the mutagenic and antimutagenic effects of Origanum compactum essential oil and some of its constituents. Mutat Res-Gen Toxen.

[B31] Nakatani N (1992). Natural antioxidants from spices.

[B32] Niture SK, Rao US, Srivenugopal KS (2006). Chemopreventative strategies targeting the MGMT repair protein: augmented expression in human lymphocytes and tumor cells by ethanolic and aqueous extracts of several Indian medicinal plants. Int J Oncol.

[B33] Özbek T, Guelluece M, Şahin F, Oezkan H, Sevsay S, Bariş Ö (2008). Investigation of the antimutagenic potentials of the methanol extract of Origanum vulgare L subsp vulgare in the Eastern Anatolia Region of Turkey. Turk J Biol.

[B34] Roofchaee A, Irani M, Ebrahimzadeh MA, Akbari MR (2013). Effect of dietary oregano (Origanum vulgare L) essential oil on growth performance, cecal microflora and serum antioxidant activity of broiler chickens. Afr J Biotechnol.

[B35] Padulosi S (1997). Oregano, Proceeding of the IPGRI International Workshop on Oregano.

[B36] Samarth R, Goyal P, Kumar A (2004). Protection of swiss albino mice against whole‐body gamma irradiation by Mentha piperita (Linn). Phytother Res.

[B37] Schmid W (1975). The micronucleus test. Mutat Res-Envir Muta.

[B38] Sharma P, Parmar J, Sharma P, Verma P, Goyal P (2011). Radiation-induced testicular injury and its amelioration by Tinospora cordifolia (An Indian medicinal plant) extract. Evid-Based Complement Alternat Med.

[B39] Sivropoulou A, Papanikolaou E, Nikolaou C, Kokkini S, Lanaras T, Arsenakis M (1996). Antimicrobial and cytotoxic activities of Origanum essential oils. J Agr Food Chem.

[B40] Teissedre P, Waterhouse A (2000). Inhibition of oxidation of human low-density lipoproteins by phenolic substances in different essential oils varieties. J Agr Food Chem.

[B41] Vicuña GC, Stashenko EE, Fuentes JL (2010). Chemical composition of the Lippia origanoides essential oils and their antigenotoxicity against bleomycin-induced DNA damage. Fitoterapia.

[B42] Whitnall MH, Elliott TB, Harding RA, Inal CE, Landauer MR, Wilhelmsen CL (2000). Androstenediol stimulates myelopoiesis and enhances resistance to infection in gamma-irradiated mice. Int J Immunopharmaco.

